# Clinical experience regarding safety and diagnostic value of cardiovascular magnetic resonance in patients with a subcutaneous implanted cardioverter/defibrillator (S-ICD) at 1.5 T

**DOI:** 10.1186/s12968-020-00626-y

**Published:** 2020-05-18

**Authors:** Viktoria Holtstiege, Claudia Meier, Michael Bietenbeck, Grigorios Chatzantonis, Anca Florian, Julia Köbe, Florian Reinke, Lars Eckardt, Ali Yilmaz

**Affiliations:** 1 Department of Cardiology I, Division of Cardiovascular Imaging, University Hospital Münster, Münster, Germany; 2 Department of Cardiology II – Electrophysiology, University Hospital Münster, Münster, Germany

**Keywords:** Implanted cardioverter/defibrillator, Cardiovascular magnetic resonance, Wide band, Late gadolinium enhancement

## Abstract

**Background:**

Cardiovascular magnetic resonance (CMR) studies in patients with implanted cardioverter/defibrillators (ICD) are increasingly required in daily clinical practice. However, the clinical experience regarding the feasibility as well as clinical value of CMR studies in patients with subcutaneous ICD (S-ICD) is still limited. Besides safety issues, image quality and analysis can be impaired primarily due the presence of image artefacts associated with the generator.

**Methods:**

Twenty-three patients with an implanted S-ICD (EMBLEM, Boston Scientific, Marlborough, Massachusetts, USA; MR-conditional) with suspected cardiomyopathy and/or myocarditis underwent multi-parametric CMR imaging. Studies were performed on a 1.5 T CMR scanner after device interrogation and comprised standard a) balanced steady state free precession cine, b) T2 weighted-edema, c) velocity-encoded cine flow, d) myocardial perfusion, e) late-gadolinium-enhancement (LGE)-imaging and f) 3D-CMR angiography of the aorta. In case of substantial artefacts, alternative CMR techniques such as spoiled gradient-echo cine-sequences and wide-band inversion-recovery LGE (wb-LGE) sequences were applied.

**Results:**

Successful CMR studies could be performed in all patients without any case of unexpected early termination or relevant technical complication other than permanent loss of the S-ICD system beeper volume in 52% of our patients. Assessment of cine-CMR images was predominantly impaired in the left ventricular (LV) anterior, lateral and inferior wall segments and a switch to spoiled gradient echo-based cine-CMR allowed an accurate assessment of cine-images in *N* = 17 (74%) patients with only limited artefacts. Hyperintensity artefacts in conventional LGE-images were predominantly observed in the LV anterior, lateral and inferior wall segments and image optimisation by use of the wb-LGE was helpful in 15 (65%) cases. Aortic flow measurements and 3D-CMR angiography were assessable in all patients Perfusion imaging artefacts precluded a meaningful assessment in at least one half of the patients. A benefit in clinical-decision making was documented in 17 (74%) patients in the present study.

**Conclusion:**

Safe 1.5 T CMR imaging was possible in all patients with an S-ICD, though the majority had permanent loss of the S-ICD beeper volume. Achieving good image quality may be challenging in some patients - particularly for perfusion imaging. Using spoiled gradient echo-based cine-sequences and wb-LGE sequences may help to reduce the extent of artefacts, thereby allowing accurate cardiac assessment. Thus, 1.5 T CMR studies should not be withhold in patients with S-ICD for safety concerns and/or fear of extensive imaging artefacts precluding successful image analysis.

## Introduction

Cardiovascular magnetic resonance (CMR) studies in patients with implanted cardioverter/defibrillators (ICD) are increasingly required in daily clinical practice. For example, identifying the presence, location and extent of myocardial inflammation and/or scars in patients with e.g. suspected myocarditis or documented ventricular tachycardia (VT), provide important information regarding clinical decision-making and risk stratification. In particular, successful identification of an ablation target in patients with e.g. ischemic or post-inflammatory scars by a preceding CMR study may increase the success of subsequent ventricular ablation procedures [1].

In the last years, many CMR studies have been performed in patients with conventional transvenous devices and there are convincing data that CMR studies can be safely performed in patients with magnetic resonance imaging (MR)-conditional devices but also in those with legacy “not tested” devices – if some precautions are considered [2, 3]. However, CMR data in patients with a subcutaneous ICDs (S-ICD) are still limited – in spite of growing clinical experience regarding the feasibility and clinical value of this device [4–7]. Besides safety issues, image quality can theoretically be impaired due to the presence of e.g. off-resonance artefacts and/or bands of signal loss caused by the S-ICD generator. It is placed on the left side of the chest with the electrode in parasternal midline, while the conventional ICD generator is usually placed in the left pectoral region below the collarbone. The S-ICD generator is thicker and larger (8.3 cm width × 6.9 cm height × 1.3 cm depth, 130 g) than the conventional transvenous ICD because of the higher energy needed for an external shock (80 joule with S-ICD vs 30–40 joule with transvenous ICD). In summary, the position next to the left main chamber and the bigger generator leads to larger metal artefacts affecting the heart. All currently commercially available S-ICDs are approved conditionally MR safe for a field strength of 1.5 T only.

In the present study, we have therefore not only assessed the safety of multi-parametric CMR studies in patients with S-ICD but have also evaluated image quality and artefact extent prior to and after applying alternative techniques for artefact reduction and image optimization. Finally, the value of CMR findings obtained in patients with S-ICD was carefully assessed with respect to clinical decision-making.

## Methods

### Study population

Between August 2017 and June 2019, 23 consecutive patients with an implanted S-ICD (EMBLEM, Boston Scientific, Marlborough, Massachusetts, USA; MR-conditional) were admitted to our CMR Centre for further diagnostic work-up of suspected ischemic/non-ischemic cardiomyopathy, myocarditis and/or ventricular arrhythmia of unknown origin by multi-parametric CMR imaging. To ensure that only patients without second active or abandoned implanted devices, components or accessories such as lead adaptors, extenders or leads were included, pre- and post-implantation chest x-rays and complete patient history were validated. Furthermore, there was no evidence of fractured electrodes or compromised pulse generator system integrity. All patients gave their written informed consent prior to the CMR study.

### CMR image acquisition

The CMR study was performed in supine position on a 1.5 T CMR scanner (Ingenia, Philips Healthcare, Best, the Netherlands) in breath-hold and with electrocardiogram (ECG)-triggering with a maximum specific absorption rate (SAR) ≤ 2 W/kg (sequence parameters, Table 1**)**. Since prominent hyperintensity artefacts in the localizer images may prevent appropriate planning of standard imaging axis, single shot (black-blood gradient-echo) acquisitions that are less prone to off-resonance artefacts could be used for planning in case of need. Balanced steady-state free precession (bSSFP)- and/or spoiled gradient echo (GRE)-based cine-CMR images were acquired in short-axis (SAx) and three long-axis (LAx) views. In addition, T2-weighted (T2w) short tau inversion-recovery (STIR) edema imaging was performed in three SAx slices. Aortic flow measurements were performed at the level of the aortic valve and/or ascending aorta using velocity-encoded cine (VENC)-CMR. Thereafter, first-pass myocardial perfusion imaging (MPI) was performed in three SAx slices (basal, mid-ventricular and apical) at resting conditions (0.075 mmol/kg body weight gadolinium (gadobutrol) was injected with a rate of 3 ml/s intravenously followed by 30 ml saline flush at the same rate) using a saturation-recovery bSSFP sequence accelerated with sensitivity encoding. Then, 3D-CMR angiography of the thoracic aorta was performed (with another infusion of 0.1 mmol/kg gadobutrol). Finally, late gadolinium enhancement (LGE)-images (T1-weighted inversion recovery gradient-echo sequence) were acquired 10–15 min after the second intravenous contrast administration in the same imaging planes as the cine-images. In case of substantial hyperintensity artefacts, alternative wide-band inversion-recovery LGE (wb-LGE) sequences were applied. Moreover, optimization of images with reduction of image artefacts was individually achieved by either acquiring images with breath-hold in inspiration, by using non-standard views and/or by changing the phase-encoding direction. VENC-CMR for aortic flow measurement, resting MPI and 3D-CMR angiography of the aorta were performed as part of our routine comprehensive CMR protocol, since an additional infusion of gadobutrol is not required if LGE-imaging is an essential part of the protocol.

**Table 1:** Sequence parameters; either automatic or volume shim was performed. For (b)SSFP sequences a SENSE factor of 2 was used

	Flip angle in °	Spatial resolution in mm^**3**^	Receiver bandwidth in MHz	TR / TE in msec	Inversion bandwidth in Hz	Acquisition time in sec
bSSFP	60	1.8 × 2.0 × 8	954	3.3 / 1.6		5.5
SSFP	15	2.0 × 2.6 × 8	1033	5.3 / 3.1		8.5
LGE	25	1.6 × 2.3 × 10	1055	6.1 / 3.0	1000	10.0
wb-LGE	25	1.6 × 2.3 × 10	1055	6.2 / 3.0	4000	10.0

*bSSFP* balanced steady state free precession; *LGE* late gadolinium enhancement; *wb-LGE* wide band

Before starting with the CMR study, the S-ICD was programmed to the MR protection mode. Hence, the VT/ventricular fibrillation (VF) therapy mode of the device was deactivated during the CMR study. Patient monitoring during the CMR examination comprised continuous ECG-, respiratory-, pulse oximetry- and blood pressure-monitoring. Following the CMR study, the device interrogation and reprogramming was repeated.

### CMR data analysis

CMR data analysis was performed off-line by two experienced readers using commercially available software (CVi42, Circle cardiovascular imaging, Calgary, Alberta, Canada). Ventricular volumes, ejection fraction and LV mass were derived by contouring endo- and epicardial borders on the short-axis cine images and indexed to body surface area (BSA). Manual delineation of the aortic lumen was performed throughout the cardiac cycle in order to obtain aortic flow values. T2w-edema, resting MPI, 3D-CMR angiography of the aorta and LGE images were visually assessed.

Assessment of image artefacts was performed visually for each CMR sequence using the following four categories: 1 = no image artefact at all and no limitation in image interpretation; 2 = good image quality with limited artefacts (affecting less than 25% of the ventricles or the target structure); 3 = poor image quality with substantial artefacts (affecting more than 25% but less than 75% of the ventricles or the target structure), and 4 = meaningful image assessment impossible due to extensive artefacts (affecting more than 75% of the ventricles or the target structure). In addition, a more detailed assessment of image artefacts was performed for cine- and LGE-imaging using a standard 17-segment model.

### Statistical analysis

Continuous variables are expressed as mean ± SD. Skewed variables are expressed as median and interquartile range (IQR). Categorical variables are expressed as frequency with percentage. Statistical analysis was performed using SPSS for Windows (version 24, Statistical Package for the Social Sciences, International Business Machines, Inc., Armonk, New York, USA). A *p*-value ≤0.05 was considered statistically significant.

## Results

### Study population

As illustrated in Table 2, the mean age of our study population was 48 ± 12 years (range 32–65 years) and 43% (*N* = 10) were women. Indications for S-ICD implantation comprised suspected ischemic (4%) and non-ischemic cardiomyopathy (22%) with a left ventricular (LV) ejection fraction (LVEF) ≤35%, suspected myocarditis and/or ventricular arrhythmias of unknown origin (74%). In the majority of our patients (65%), S-ICD implantation was performed for secondary prophylactic reasons.

**Table 2:** Baseline patient characteristics

Parameter	Value
Age, years	48 ± 12
Gender male/female, n (%)	13 (57%) / 10 (43%)
Height, cm	179 ± 9
Weight, kg	87 ± 18
BMI, kg/m^2^	27 ± 4
BSA, m^2^	2.0 ± 0.2
NYHA class I / II / III / IV, n (%)	11 (48%) / 5 (22%) / 3 (13%) / 0 (0%)
Indication for S-ICD implantation, n (%)	
Suspected non-ischemic cardiomyopathy with LVEF ≤35%	5 (22%)
Suspected ischemic cardiomyopathy with LVEF ≤35%	1 (4%)
Ventricular arrhythmia of unknown origin	17 (74%)
Primary vs. secondary prophylaxis, n (%)	8 (35%) vs. 15 (65%)

*BMI* body mass index; *S-ICD* subcutaneous implanted cardioverter/defibrillator; *LVEF* left ventricular ejection fraction

### Safety issues

A successful CMR study could be performed in all patients without any case of unexpected early termination being required due to technical and/or vital complications **(**Table 3**)**. No power reset, battery discharge, reprogramming, neurological symptoms or thermal sensation were observed. Lead impedance and function were unaffected in all patients after the scan. The only technical issue that was observed during this study, was a complete loss of the S-ICD beeper volume in 52% of subjects after the CMR study (after having contact with the magnetic field). During the CMR study, premature ventricular complexes were monitored in 35% of our patients – without any need to change the study protocol.

**Table 3:** Device characteristics and safety issues

Parameter	Value
MR-conditional, n (%)	23 (100%)
S-ICD device model A 209, n (%)	23 (100%)
Electrode model, n (%)	
3401	12 (52%)
3501	9 (39%)
3010	2 (9%)
Safety issues associated with CMR, n (%)	
Death	0 (0%)
Device replacement	0 (0%)
Electrical reset	0 (0%)
Change of impedance	0 (0%)
Change in battery voltage	0 (0%)
VT/VF/hemodynamic instability	0 (0%)
PVC during CMR	8 (35%)
Loss of device alarm	12 (52%)

*MR* magnetic resonance; *PVC* premature ventricular complex; *VF* ventricular fibrillation; *VT* ventricular tachycardia

### CMR image artefacts and findings in S-ICD patients

The detailed results regarding assessment of CMR image quality and artefact extent for each CMR sequence used in the present study are given in Table 4. Notably, there was no relevant artefact caused by the subcutaneous electrode of the S-ICD system in parasternal midline in any CMR sequence used in the present study.

**Table 4:** CMR pulse-sequence details and image quality assessment

Parameter	Value
Used type of cine-sequence	
Steady-state free precession (SSFP), n (%)	9 (39%)
Spoiled gradient echo (GRE), n (%)	23 (100%)
Artefacts in cine images after optimization, n (%)	
No artefacts / no limitation	0 (0%)
Good image quality with limited artefacts	17 (74%)
Poor image quality with substantial artefacts	5 (22%)
Image assessment impossible due to extensive artefacts	1 (4%)
Used type of LGE-sequence	
Conventional inversion-recovery LGE-sequence, n (%)	22 (96%)
Modified wide-band LGE-sequence, n (%)	20 (87%)
Artefacts in LGE images after optimization	
No artefacts / no limitation	0 (0%)
Good image quality with limited artefacts	13 (56%)
Poor image quality with substantial artefacts	10 (44%)
Image assessment impossible due to extensive artefacts	0 (0%)
Artefact minimization by additional use of wide-band LGE, n (%)	15 (65%)
Artefact minimization by changing phase encoding direction, n (%)	9 (39%)
T2-weighted edema imaging, n (%)	23 (100%)
Artefacts in T2-weighted edema images, n (%)	
No artefacts / no limitation	1 (4%)
Good image quality with limited artefacts	15 (65%)
Poor image quality with substantial artefacts	7 (30%)
Image assessment impossible due to extensive artefacts	0 (0%)
Velocity-encoded phase-contrast imaging of aortic flow, n (%)	21 (91%)
Artefacts in phase-contrast aortic flow images, n (%)	
No artefacts / no limitation	14 (61%)
Good image quality with limited artefacts	7 (30%)
Poor image quality with substantial artefacts	0 (0%)
Image assessment impossible due to extensive artefacts	0 (0%)
Myocardial perfusion imaging (MPI) at rest, n (%)	16 (70%)
Artefacts in resting perfusion images, n (%)	
No artefacts / no limitation	1 (4%)
Good image quality with limited artefacts	7 (30%)
Poor image quality with substantial artefacts	5 (22%)
Image assessment impossible due to extensive artefacts	3 (13%)
3D-CMR angiography of the aorta, n (%)	15 (65%)
Artefacts in 3D-CMR angiography images, n (%)	
No artefacts / no limitation	14 (61%)
Good image quality with limited artefacts	1 (4%)
Poor image quality with substantial artefacts	0 (0%)
Image assessment impossible due to extensive artefacts	0 (0%)

**Fig. 1:**
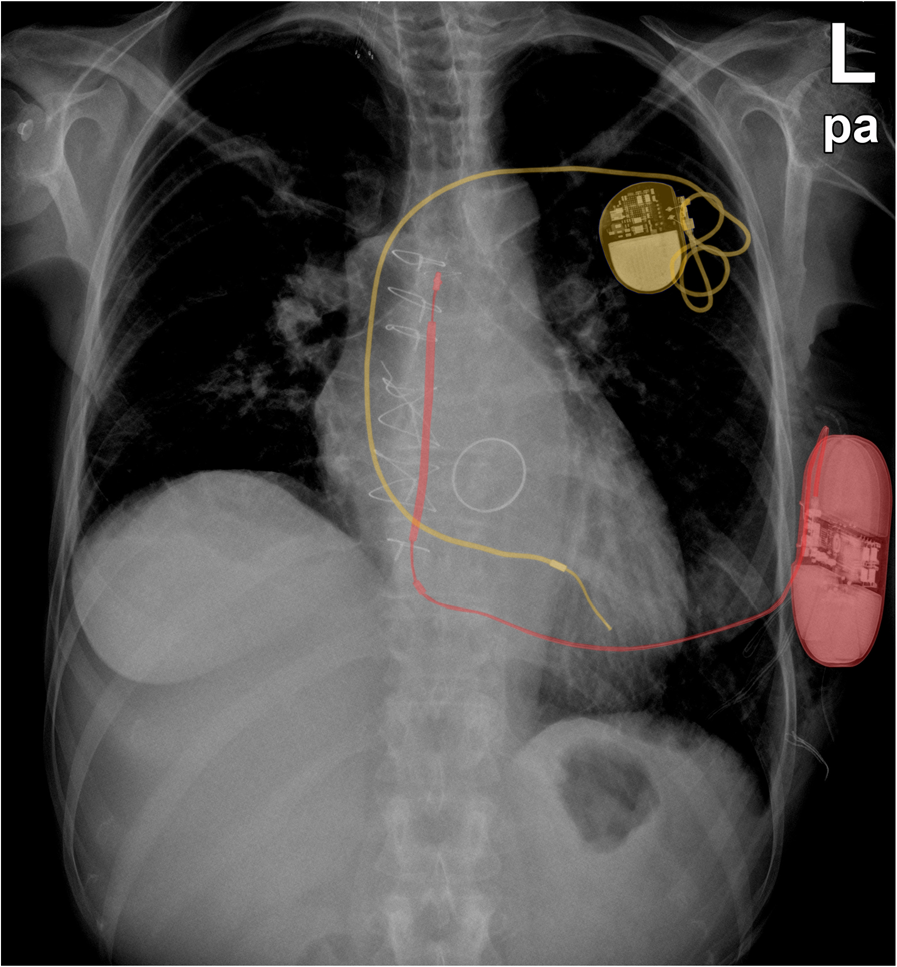
Chest x-ray of a patient carrying both, a conventional device (pacemaker (yellow)) and an S-ICD (red)

**Fig. 2:**
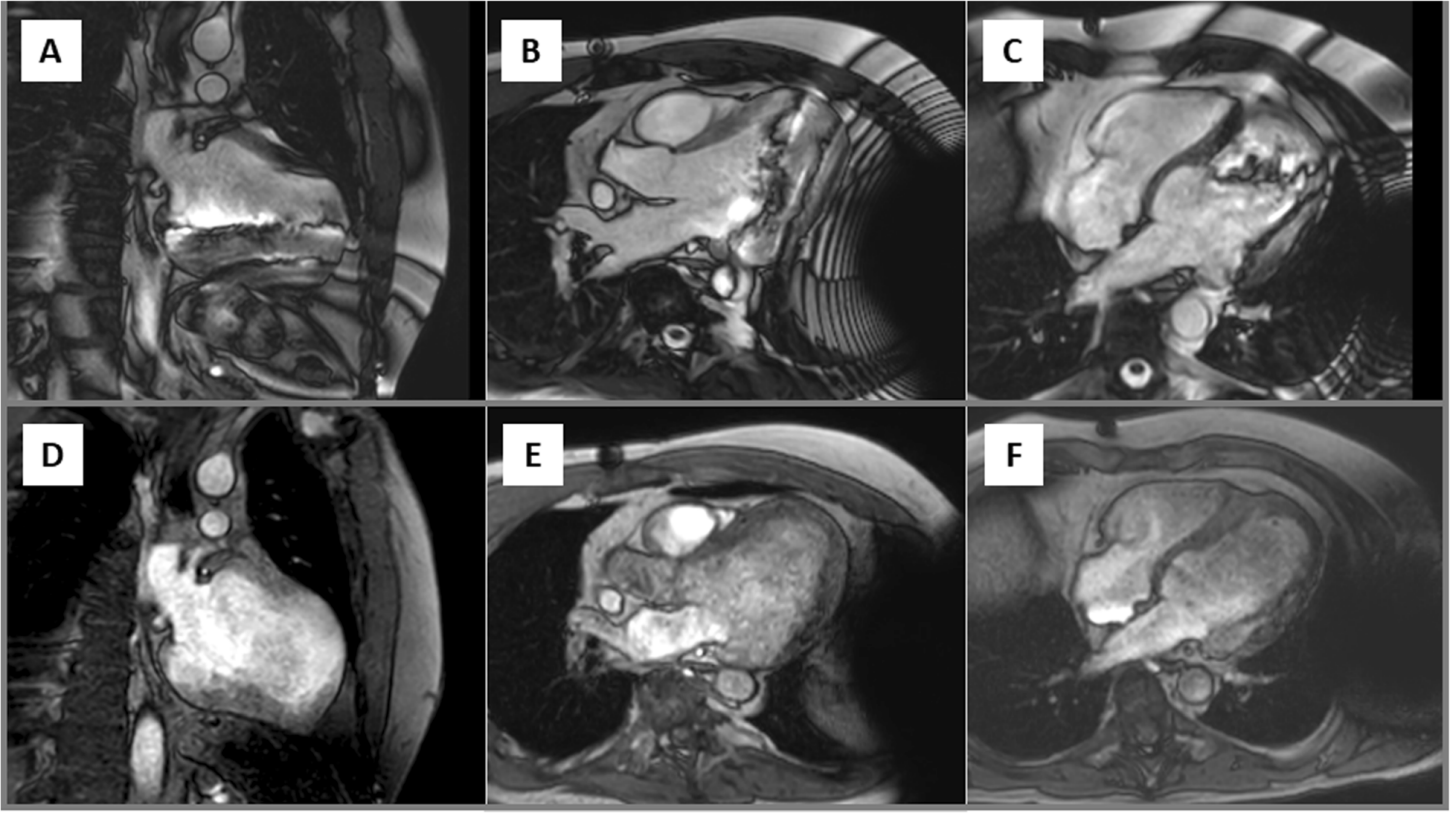
Cine-cardiovascular magnetic resonance (CMR) images in a patient with ischemic cardiomyopathy and extensive anterior scar illustrating the extent of the generator artefact. Balanced steady-state free precession (bSSFP)-based cine-images in a left ventricular (LV) 2-chamber (**a**), 3-chamber (**b**) and 4-chamber view (**c**) with artefacts in the inferolateral and apical wall segments. After switching to spoiled gradient echo (GRE)-based cine-CMR the aforementioned artefacts disappeared completely in this patient without any relevant artefact in the region of interest

**Fig. 3:**
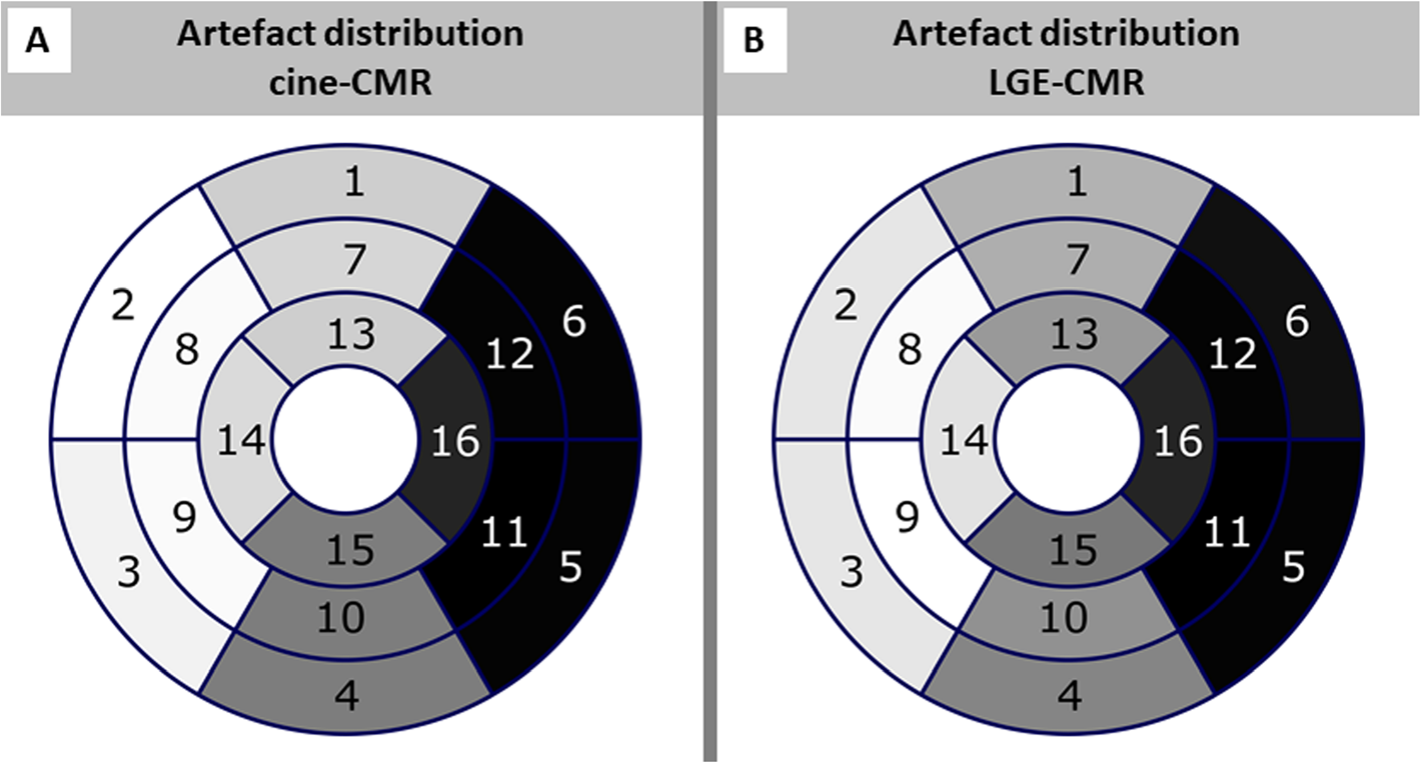
Bulls-eye plot visualizing average artefact distribution in cine-CMR (**a**) and late gadolinium enhancement (LGE)-CMR (**b**) images (grey scale with white color = excellent assessability and black = very poor assessability)

Due to the location of the S-ICD generator **(**Fig. 1**)**, assessment of bSSFP-based cine-CMR images was predominantly impaired in the LV anterior, lateral and inferior wall segments **(**Fig. 2a-c**)**. However, a switch to spoiled GRE-based cine-CMR substantially improved image quality in the majority of cases **(**Fig. 2d-f**)** and allowed an accurate assessment of cine-images in *N* = 17 (74%) patients with only limited artefacts. There was only one study in which assessment of cine-images was completely impossible due to very extensive device generator artefacts. The overall distribution of artefacts in cine-images is further illustrated in Fig. 3a.

**Fig. 4:**
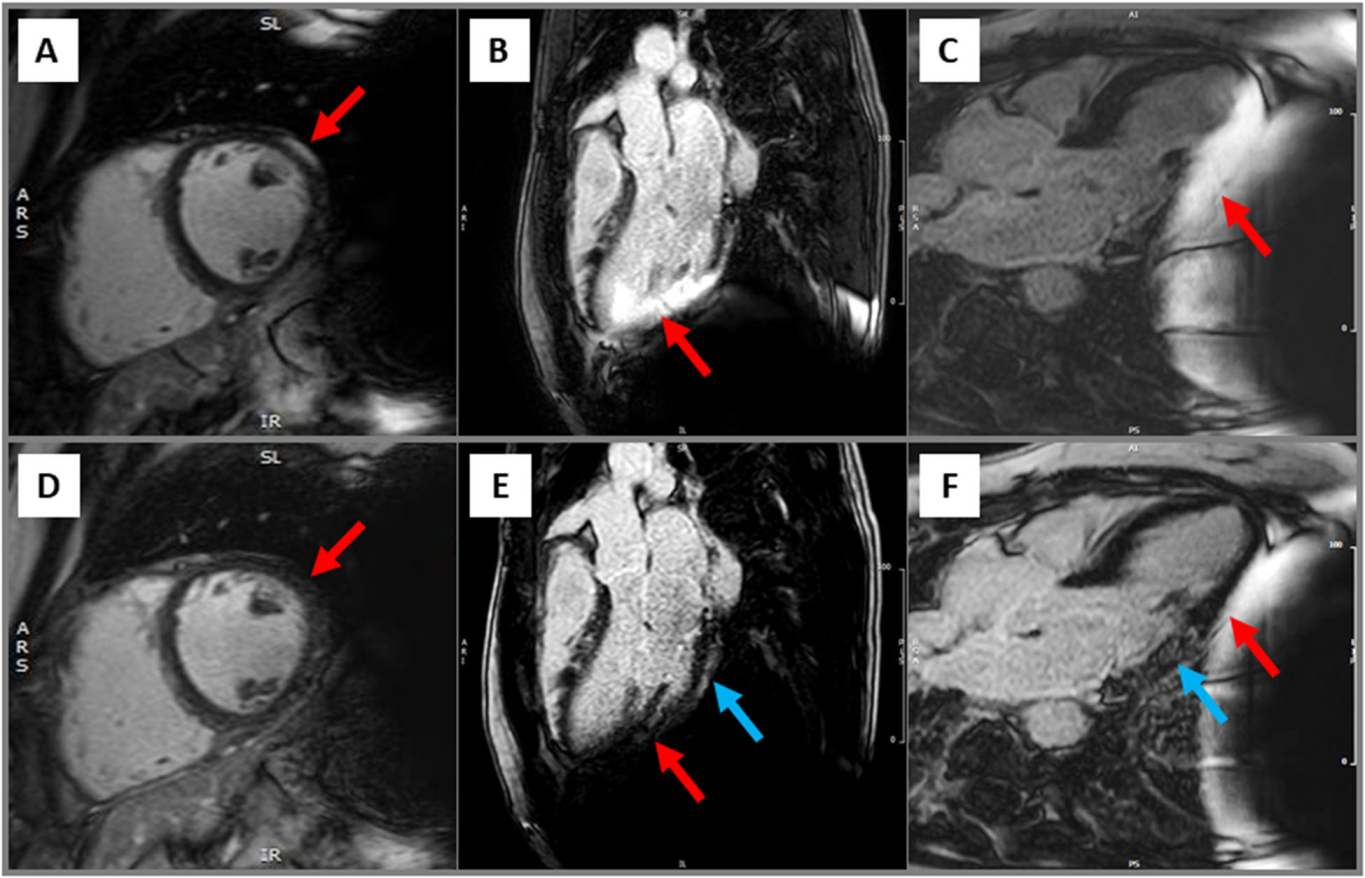
Hyperintensity artefact (red arrow) suggesting an epicardial scar in the anterolateral wall segment (**a**) in a patient without LGE as shown in the same view after image optimisation by the wide-band LGE (wb-LGE) technique (**d**). Hyperintensity artefact suggesting an ischemic pattern of myocardial damage in the inferolateral wall (**b**, red arrow); disappearance of this artefact and unmasking of an epicardial LGE using the wb-LGE technique (**e**, blue arrow). Minimization of the hyperintensity artefact in the inferolateral wall segments (**c**, red arrow) by inspiration and unmasking of an intramural LGE (**f**, blue arrow)

Similar to cine-images, hyperintensity artefacts in conventional LGE-images were predominantly observed in the LV anterior, lateral and inferior wall segments caused by the S-ICD generator **(**Fig. 3b **and** Fig. 4a-c**)**. Using optimized methods, LGE images with only limited artefacts were obtained in 13 (56%) patients. Noteworthy, the septal wall was assessable in all but one case, but poor image quality with substantial artefacts were documented in 10 (44%). Image optimisation by the wide-band technique was helpful in 15 (65%) of cases **(**Fig. 4d-f**)** and/or changing the phase encoding direction improved image quality in 9 (39%) patients.

**Fig. 5:**
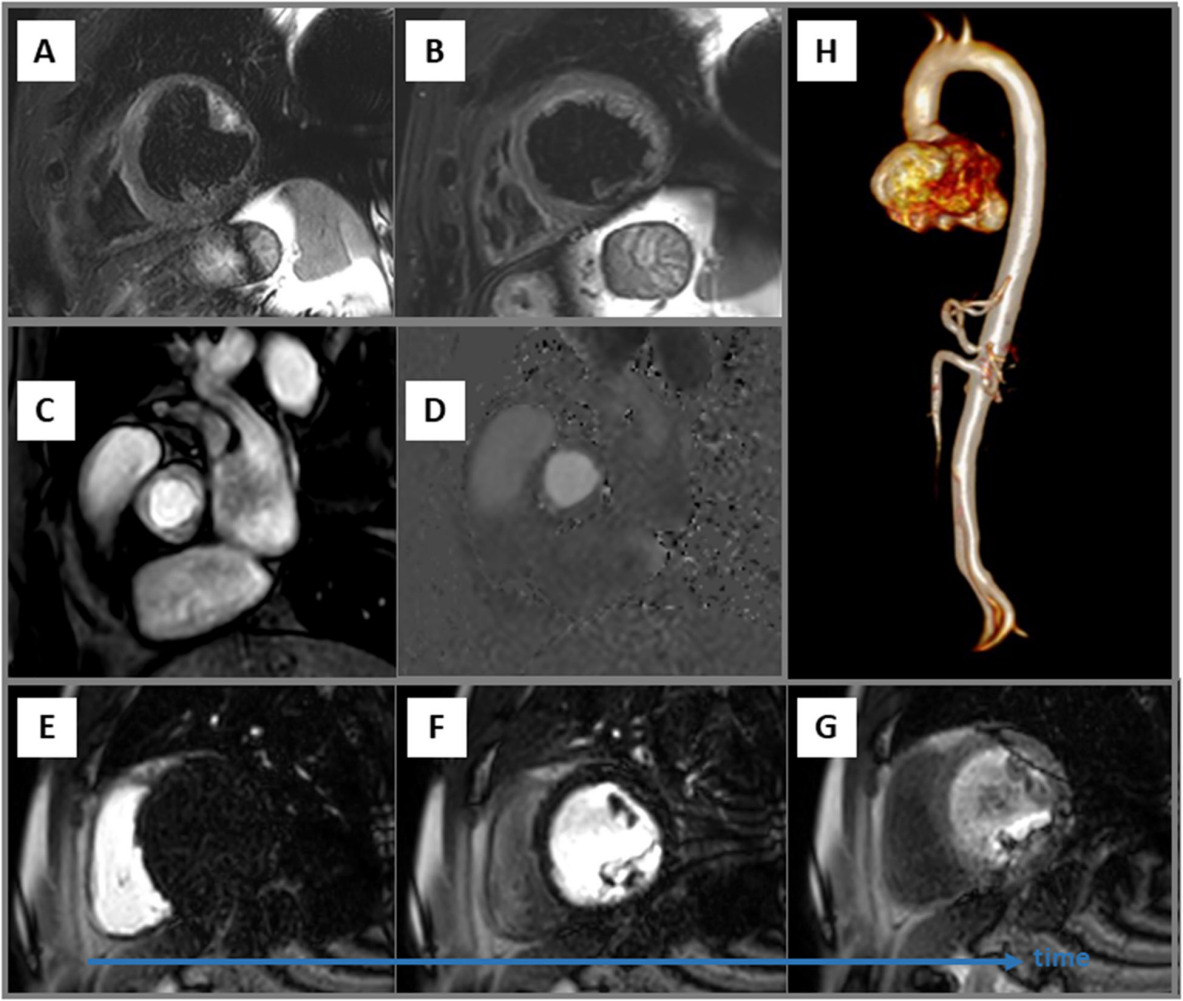
Examples of short-axis views of T2-weighted images (**a-b**) without any relevant device artefact in the region of interest. Artefact-free anatomic (**c**) and phase-contrast flow images (**d**) of the aortic valve performed to assess aortic flow by velocity encoding (VENC) CMR. Short-axis perfusion images at different time-points with illustrating substantial image artefacts in the anterolateral and inferolateral wall segments that preclude a valid visual assessment in these segments (**e-g**). Example of an artefact-free reconstruction of 3D-CMR angiography of the thoracic aorta (H)

Regarding T2w edema imaging, good image quality without any or only limited artefacts was observed in 16 (70%) patients **(**Fig. 5a-b**)**. Aortic flow measurements using VENC-CMR was performed in 21 (91%) patients and was assessable without any relevant artefact issue in all of them **(**Fig. 5c-d**)**. First-pass MPI at rest was performed in  16 (70%) patients and only half of these patients demonstrated perfusion images with acceptable image quality allowing valid visual assessment **(**Fig. 5e-g**)**. Finally, additional 3D-CMR angiography of the thoracic aorta was performed in 15 (65%) patients and artefact-free reconstruction and assessment of the thoracic aorta was possible in all of them (Fig. 5h).

The major CMR findings in the present study are illustrated in Table 5. The average LVEF was 58 ± 10% whereas mean right ventricular (RV) ejection fraction (RVEF) was 58 ± 9%. An ischemic pattern of LGE was observed in 1 (4%) patient whereas a non-ischemic pattern of myocardial fibrosis was documented in 16 (70%); in 4 (22%) patients, the LGE pattern (in addition to T2w edema imaging) was suggestive of an inflammatory cardiomyopathy (including myocarditis and cardiac sarcoidosis).

**Table 5:** CMR findings

Parameter	Value
Left ventricular CMR findings	
LVEF, %	58 ± 10%
LV EDV, ml	197 ± 68
LV ESV, ml	90 ± 52
LV mass, g	162 ± 42
Right ventricular CMR findings	
RVEF, %	58 ± 9%
RV EDV, ml	180 ± 49
RV ESV, ml	77 ± 32
LGE findings (after image optimization), n (%)	
No LGE	6 (26%)
Presence of LGE with ischemic pattern	1 (4%)
Presence of LGE with non-ischemic pattern	16 (70%)
Extent of LGE, no. of segments	3 ± 3
Extent of LGE, % of total myocardial mass	20 ± 17%
CMR-based final diagnosis, n (%)	
Ischemic cardiomyopathy	1 (4%)
Non-ischemic cardiomyopathy	14 (61%)
Myocarditis/inflammatory cardiomyopathy	5 (22%)
Normal CMR findings/non-conclusive CMR	3 (13%)

*EDV* end-diastolic volume; *ESV* end-systolic volume; *RV* right ventricular; *RVEF* right ventricular ejection fraction

### Value of CMR data regarding clinical decision-making

A benefit in clinical decision-making was defined as illustrated in Table 6 and comprised (amongst others) a) identifying a new or confirming/changing a suspected diagnosis, b) identifying and locating myocardial scarring and assessing transmurality (helpful for optimal planning of ablation procedures and for risk stratification, e.g. assessment of the potential for future full functional recovery), c) identifying and locating myocardial inflammation for appropriate diagnosis of inflammatory diseases and subsequent targeted endomyocardial biopsy as well as therapy and d) targeted genetic analysis in those cases in which CMR was suggestive of a genetic cardiomyopathy (one patient with suspected cardiac laminopathy and another one with arrhythmogenic RV cardiomyopathy (ARVC) in the present study). Taken together, a benefit in clinical-decision making was documented in 17 (74%) patients in the present study.

**Table 6:** Value of CMR findings in clinical decision-making

Parameter	Value
Benefit from CMR for clinical decision-making, n (%)	
Yes	17 (74%)
No	6 (26%)
Details of clinical decision-making based on CMR findings, n (%)	
Conclusive diagnosis of underlying cardiac disease in so far unclear condition	8 (35%)
Change in previously suspected cardiac diagnosis	3 (13%)
Identification of myocardial scar location for targeted ablation	1 (4%)
Change in cardiac medication	2 (9%)
Targeted genetic analysis based on CMR findings	2 (9%)
Additional cardiac work-up in family members due to CMR findings	2 (9%)
Optimized scheduling of cardiac follow-up studies	7 (30%)
Targeted endomyocardial biopsy based on CMR findings	3 (13%)

## Discussion

CMR studies in patients with ICDs are increasingly required in daily clinical practice. However, the clinical experience regarding the feasibility, limitations and clinical value of CMR studies in patients with S-ICDs remains limited. To the best of our knowledge, the present study is the first that has evaluated not only safety issues of CMR studies in S-ICD patients but also addressed image quality and subsequent benefit in clinical decision-making. The major results of the present study show a) that safe and high-quality CMR imaging is possible in patients with implanted S-ICD, b) that using spoiled GRE-based cine-sequences and wb-LGE sequences may tremendously help to reduce the extent of artefacts, thereby allowing accurate cardiac assessment and c) that the information obtained from such CMR studies is mostly helpful in clinical decision-making.

Due to the technical progress achieved in the last years, the presence of an ICD system is no longer an absolute contraindication for CMR studies and comprehensive CMR studies are even reasonable for patients with a legacy/“non-tested” (non-conditional) ICD system [2, 3]. However, after almost a decade since the introduction of the first S-ICD generation (SQ-RXVR 1010; Cameron Health now Boston Scientific) in 2009, the clinical experience regarding CMR studies is still limited [8, 9]. Current S-ICD generators (EMBLEM S-ICD, EMBLEM MRI S-ICD, Model A209, A219 of Boston Scientific) and all models of electrodes (Boston Scientific EMBLEM S-ICD Electrode, Cameron Health Q-TRAK S-ICD Electrode) are components of the ImageReady S-ICD system that is principally approved for use with 1.5T MR scanners - under the conditions of use described in the technical guide [10] – and for any part of the body.

In the present study, CMR could be performed safely in all patients and there was no S-ICD system malfunction or any other relevant complication in spite of the focus of the magnetic field and the radiofrequency energy being directly over the area of the S-ICD device generator. Noteworthy, the beeper/alarm noise of the S-ICD system (that is signalling audible tones in case of elective replacement time and end-of-life, out-of-rage electrode impedance, prolonged charging time and irregular battery depletion) was affected in the majority of patients. This is in line with previous reports on intravenous ICD systems of Boston Scientific. Interestingly, it turned out that only in about one half of the patients (52%) the alarm noise was lost – without any further technical changes being observed. To our surprise, the beeper function was re-activated after a second follow-up CMR study in one of our patients. For our patients the loss of the beeper volume does not represent a major safety issue, since all patients are checked in a 90-day time interval in our outpatient clinic and/or have a LATITUDE NXT Wave Communicator for telemetric follow-up. Moreover, ICD alarms emitted in outpatient settings are very rarely recognized by the patients and vibratory alarm seems to be more effective compared to audible alerts [11].

In principle, CMR image artefacts in device patients are associated with a) the size of the device generator and b) its distance to the heart [12, 13]. Additional file 1: Figure 6 illustrates the extent of metal artefacts caused by the generator in case of a conventional transvenous ICD compared to a S-ICD. Hence, the usual size and location of the S-ICD generator (next to the LV apex) are rather unfavourable for high quality CMR imaging. In more detail, the bSSFP-cine sequence that is most commonly used for assessment of cardiac function and anatomy is characterized by both an excellent blood-myocardium contrast and a low sensitivity to the inflow of unsaturated blood. On the other hand, this sequence is highly susceptible to magnetic field inhomogeneities, e.g. caused by metallic devices such as the S-ICD generator **(**Additional file 2: **Figure 7)**. Therefore, spoiled GRE-based sequences are more suitable for CMR in case of cardiac device patients, since they are less susceptible to such field inhomogeneities and subsequent artefacts [14]. In our S-ICD population, use of the spoiled GRE-based sequence was mostly helpful in reducing artefacts in cine-images and substantially improved image quality – at the expense of a reduced contrast-to-noise (CNR) ratio. In a few patients, some signal loss remained even when using spoiled GRE-based sequences in the LV lateral and anterior wall segments due to the generator’s proximity.

Today, LGE-imaging is the gold standard for non-invasive CMR-based assessment of myocardial fibrosis/scar and viability. Conventional LGE-sequences are based on inversion-recovery pulses that are applied in order to null the signal from healthy myocardium and thereby to improve the visibility of contrast-enhanced damaged myocardial tissue. However, in the surrounding of metallic devices, a strong frequency off-resonance artefact may occur depending on the exact location and size of the respective device. It has been shown recently that a cardiac device in 5–10 cm distance to the heart usually results in a magnetic resonance frequency offset of 2–6 kHz in the myocardium at 1.5-T [15]. As recently shown in patients with conventional cardiac devices [13, 15–23], we have used an adiabatic wide-band inversion-recovery pulse with an adjustable frequency offset to improve the myocardial nulling even in the presence of off-resonance effects. In the majority of our LGE-images (56%), the respective images showed sufficient quality for a meaningful diagnostic assessment. Even in those cases with low quality and/or extensive artefacts, visualization of only a few artefact-free segments allowed to detect the presence of LGE and to suggest a diagnosis. Therefore, even the presence of off-resonance artefacts in a substantial number of myocardial segments does not inevitably preclude a conclusive diagnosis based on LGE-imaging.

Obviously, the assessment of the value of imaging data for clinical-decision making is quite challenging and somewhat arbitrary since clinical-decision making per se can be rather subjective and does not necessarily reflect a standardized approach. Nevertheless, we made some efforts in order to comprehensively determine the respective benefit of CMR data in clinical decision-making as illustrated in detail in Table 6. Noteworthy, a conceivable benefit for clinical decision-making has been observed in the majority (74%) of our patients with S-ICD – primarily based on spoiled GRE-based cine-, T2w edema and wb-LGE-imaging.

In addition to cine-, T2w edema and LGE-imaging, we also performed VENC for aortic flow measurement, resting MPI and 3D-CMR angiography of the aorta in the majority of our patients with implanted S-ICDs. Whereas aortic flow measurements were assessable without any relevant artefact issue in all of our patients (due to the location of the ascending aorta in a sufficient distance from the S-ICD generator), MPI using a bSSFP sequence was challenging and image artefacts precluded a meaningful assessment in at least one half of the patients. Hence, improved protocols for perfusion imaging (such as wide-band myocardial perfusion pulse sequence [23] or optimized saturation-recovery GRE sequences) are required before stress-perfusion protocols can be suggested without restrictions in S-ICD patients.

Aortic angiography is often performed and frequently repeated in adults with congenital heart disease or Marfan’s syndrome. CMR imaging is principally preferred to computed tomography (CT) due to the lack of any ionizing radiation and iodinated contrast burden with CMR. In this context, 3D-CMR angiography in the present study showed an unrestricted good image quality without any relevant artefacts. Analysis of aortic diameter and anatomy could be performed accurately. Noteworthy, the present study is the first one that illustrates excellent image quality of 3D-CMR angiography in patients with an S-ICD.

A general statement and/or recommendation whether CMR in patients with S-ICD is “more” or “less” prone to imaging artefacts compared to conventional transvenous ICD devices is not possible, since the relevance and extent of such metal artefacts are mainly determined by the distance of the device to the heart [13, 16]. Obviously, this distance is dependent on individual patient constitution and location of the respective device. Theoretically, the usual location of the S-ICD generator - next to the LV apex - is rather unfavourable for CMR imaging **(**Additional files 1 and 2: Figures 6 and 7**)**. However, in case of a transvenous ICD device positioned rather next to the heart (at the level of the atria), the respective metal artefacts caused by the generator will be tremendously more relevant compared to a rather subclavicular location in a very tall patient. Unfortunately, the present study size was too small to derive any meaningful distance values for S-ICDs. This important issue will be addressed in future studies with larger study size.

### Study limitations

One limitation of our study is the relative small number of patients. However, to the best of our knowledge the present study comprises the world’s largest S-ICD population undergoing CMR so far. The major goal of this study was to illustrate the feasibility, safety and clinical value of CMR in patients with a S-ICD. Future work needs to consider larger study groups and further upcoming imaging techniques such as T1- and/or T2-mapping. Finally, some previous studies suggested a correlation between minimal generator-heart distance and artefact size on LGE-images for patients with transvenous devices [13, 16]. Such a conceivable association could not be confirmed in the present study – probably due to the small study group.

## Conclusion

Safe 1.5 T CMR imaging was possible in all patients with implanted S-ICD though over half had permanent loss of the S-ICD system beeper volume. Achieving good image quality may be challenging in some patients - particularly in case of MPI. Using spoiled GRE-based cine-sequences and wb-LGE sequences may help to reduce the extent of artefacts, thereby allowing accurate cardiac assessment. Thus, 1.5 T CMR studies should not be principally withhold in patients with S-ICD for safety concerns and/or fear of extensive imaging artefacts precluding successful image analysis.

## Supplementary information

**Additional file 1: Figure 6.** Illustration of the extent of metal artefacts caused by a conventional ICD vs. a S-ICD in relation to their location/distance to the heart. A shorter distance between the respective device and the heart results in more artefacts in the region of interest

**Additional file 2: Figure 7.** (A) Survey images taken from a water phantom with either an ICD or S-ICD attached to the bottom, showing the larger artifacts produced by the S-ICD; (B) respective B0-maps and corresponding line plots showing the induced frequency shifts to the baseline frequency – noteworthy: from zero to peak frequency shift (ICD approx. 6 cm, S-ICD approx. 9 cm) the induced shift is greyed out/nulled due to too high B0-field disturbances. For example, at a distance of 12.5 cm between heart and device, the S-ICD will induce an approx. 700 Hz larger frequency shift than a conventional ICD.

## Data Availability

The datasets used and/or analysed during the current study are available from the corresponding author on reasonable request.

## Abbreviations

ARVC: Arrhythmogenic right ventricular cardiomyopathy
ATP: Antitachycardia pacing
BMI: Body mass index
BSA: Body surface area
bSSFP: Balanced steady-state free precession
CMR: Cardiovascular magnetic resonance
CNR: Contrast-to-noise
CRT: Cardiac resynchronisation therapy
CT: Computed tomography
ECG: Electrocardiogram
EDV: End-diastolic volume
ESV: End-systolic volume
GRE: Gradient echo
ICD: Implanted cardioverter/defibrillator
IQR: Interquartile range
LAx: Long axis
LGE: Late gadolinium enhancement
LV: Left ventricle/left ventricular
LVEF: Left ventricular ejection fraction
MAVERIC: Multi-acquisition variable resonance image combination
MPI: Myocardial perfusion imaging
MR: Magnetic resonance
ns-VT: Non sustained ventricular tachycardia
NYHA: New York Heart association
PVC: Premature ventricular complexes
RV: Right ventricle/right ventricular
RVEF: Right ventricular ejection fraction
SAx: Short axis
SAR: Specific absorption rate
SEMAC: Slice encoding for metal artefact compensation
S-ICD: Subcutaneous implantable cardioverter/ defibrillator
STIR: Short tau inversion-recovery
T2w: T2 weighted
VENC: Velocity encoded cine
VENC: Velocity encoded
VF: Ventricular fibrillation
VT: Ventricular tachycardia
wb-LGE: Wideband late gadolinium enhancement
