# Supplementary figures and images for: Clinical experience regarding safety and diagnostic value of cardiovascular magnetic resonance in patients with a subcutaneous implanted cardioverter/defibrillator (S-ICD) at 1.5 T
Source: J Cardiovasc Magn Reson. 2020 May 18;22:35. doi: 10.1186/s12968-020-00626-y (PMC7232845; doi:10.1186/s12968-020-00626-y)

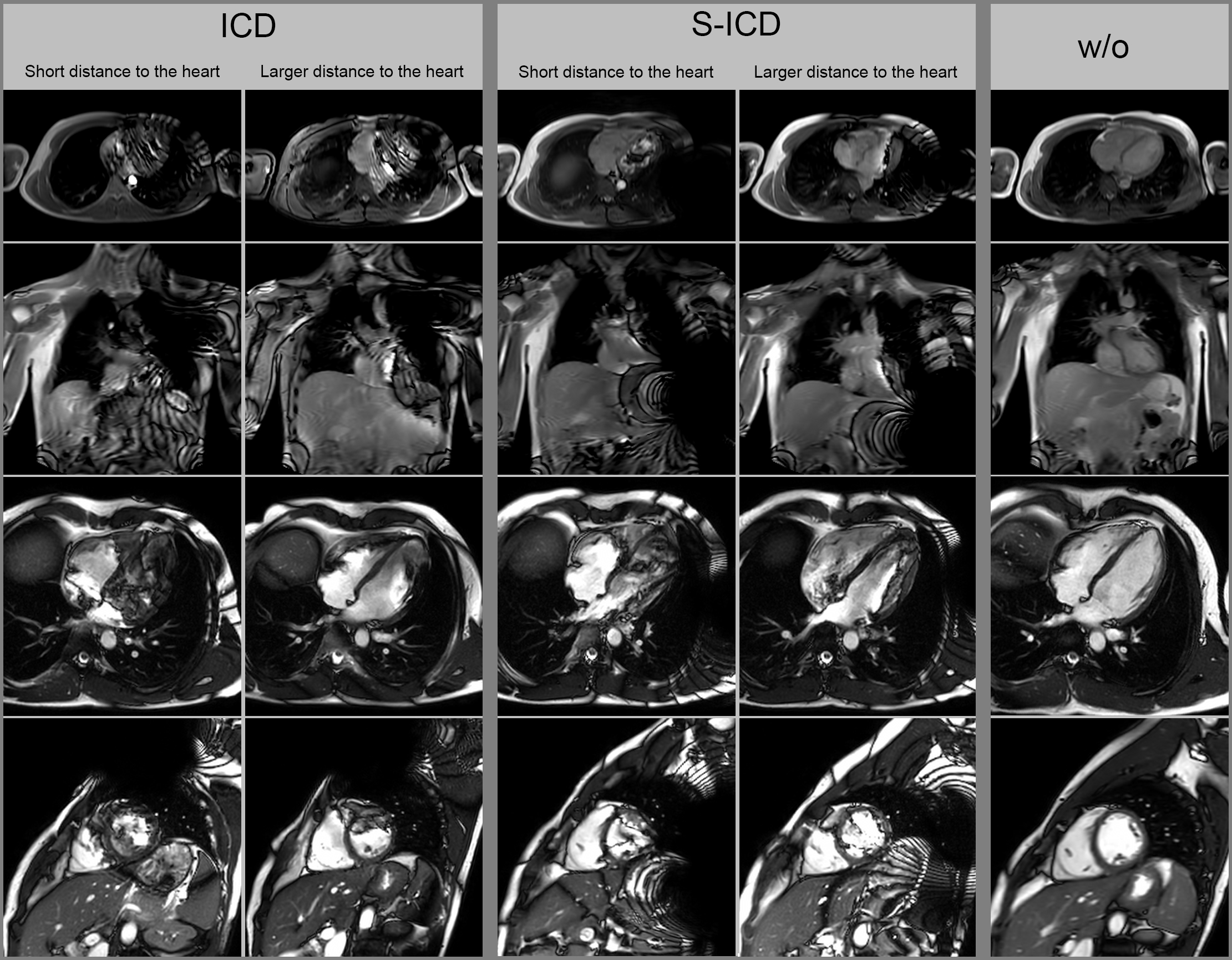

Supplement: Supplementary file 1 — Additional file 1: Figure 6. Illustration of the extent of metal artefacts caused by a conventional ICD vs. a S-ICD in relation to their location/distance to the heart. A shorter distance between the respective device and the heart results in more artefacts in the region of interest [file 12968_2020_626_MOESM1_ESM.jpg]

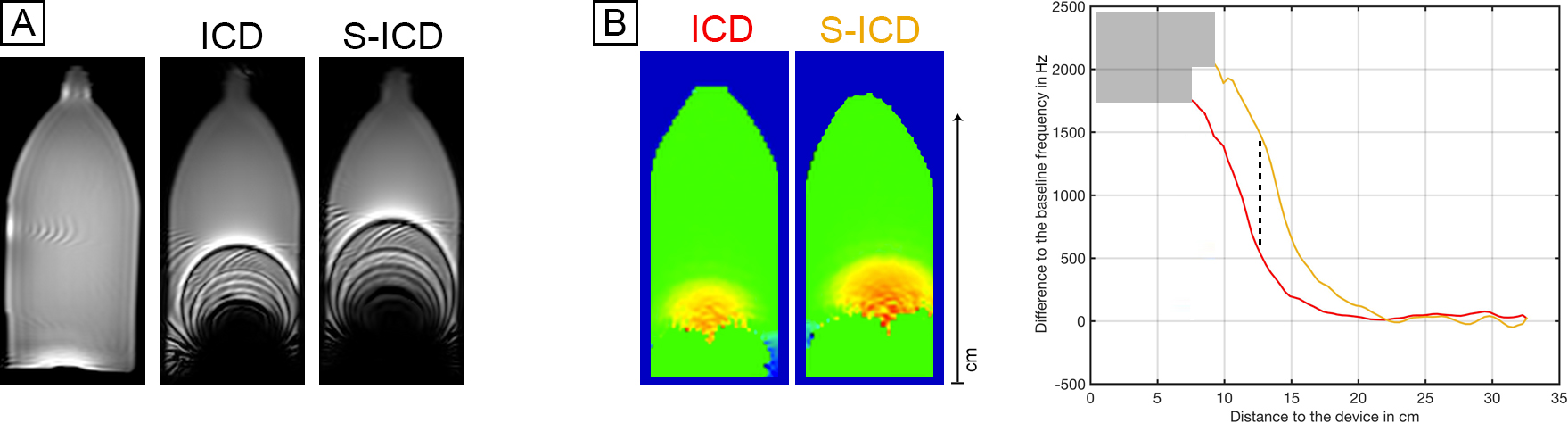

Supplement: Supplementary file 2 — Additional file 2: Figure 7. (A) Survey images taken from a water phantom with either an ICD or S-ICD attached to the bottom, showing the larger artifacts produced by the S-ICD; (B) respective B0-maps and corresponding line plots showing the induced frequency shifts to the baseline frequency – noteworthy: from zero to peak frequency shift (ICD approx. 6 cm, S-ICD approx. 9 cm) the induced shift is greyed out/nulled due to too high B0-field disturbances. For example, at a distance of 12.5 cm between heart and device, the S-ICD will induce an approx. 700 Hz larger frequency shift than a conventional ICD. [file 12968_2020_626_MOESM2_ESM.jpg]
